# Experiences and perspectives of parents on their care roles in two Rwandan public neonatal units: a qualitative study

**DOI:** 10.11604/pamj.2025.51.94.32507

**Published:** 2025-08-14

**Authors:** Agnes Mukaruziga, Aimable Musafili, Peter Cartledge

**Affiliations:** 1 Department of Pediatrics and Child Health, School of Medicine and Pharmacy, University of Rwanda, Kigali, Rwanda,; 2 Department of Pediatrics, University Teaching Hospital of Kigali (CHUK), Kigali, Rwanda,; 3 Department of Pediatrics, University Teaching Hospital of Butare (CHUB), Butare, Rwanda,; 4 Human Resources for Health (HRH) Program, Ministry of Health, Kigali, Rwanda,; 5 Department of Emergency Medicine, Yale University, New Haven, Connecticut, USA

**Keywords:** Intensive care unit, neonatal, infant, preterm, parents, caregivers

## Abstract

**Introduction:**

family integrated care (FICare) is increasingly regarded as best practice in neonatal units. However, little is known about how parents experience and perceive their care roles in neonatal units in low-resource settings such as Rwanda. This study explored lived experiences, attitudes, and perceptions of caregivers regarding parental care roles in two neonatal units in Rwanda.

**Methods:**

a qualitative study using semi-structured face-to-face interviews was conducted in one tertiary and one secondary neonatal unit in Kigali, Rwanda. A pragmatic content analysis approach was applied without a guiding theoretical framework.

**Results:**

sixteen caregivers (14 mothers, 1 father, 1 aunt) were interviewed; their infants had a mean gestational age of 29.5 weeks (SD±1.45). Seven themes emerged: experiences in the neonatal unit, current caregiver roles, readiness to provide care, attitudes towards extended roles, perceived benefits, barriers, and support needs. Parents reported emotional distress related to their infants´ illnesses and the hospital setting. They were already engaged in tasks such as feeding, hygiene, and bonding. Most expressed willingness to take on more responsibilities if supported through education and guidance. Reported barriers included limited skills, maternal illness, financial constraints, and restricted access to infants due to hospital policies and infrastructure.

**Conclusion:**

parents in two Rwandan neonatal units expressed a strong desire to participate more actively in their newborns' care. Efforts to expand their care roles should be encouraged and supported, where possible and safe, through structured education, accessible infrastructure, and emotional and material support from healthcare professionals.

## Introduction

Neonatal mortality remains a critical global health challenge, particularly in sub-Saharan Africa. In 2018, 2.5 million neonatal deaths were recorded worldwide. While the global neonatal mortality rate was estimated at 18 deaths per 1,000 live births, sub-Saharan Africa reported a much higher rate of 78 per 1,000 live births [1,2]. In response, the international community committed to Sustainable Development Goal 3 (SDG 3), which seeks to reduce neonatal mortality to 12 deaths per 1,000 live births by 2030 [3]. In Rwanda, the neonatal mortality rate declined from 50 to 32 per 1,000 live births between 2010 and 2015. However, the current rate of 20 per 1,000 live births highlights the need for sustained action to achieve this global target [4]. Over the past five decades, significant advances in neonatal care have enabled the survival of infants previously considered nonviable. Despite these improvements, the hospitalization of newborns remains a distressing experience for parents, who often report feelings of helplessness, fear, and emotional strain when navigating unfamiliar neonatal unit environments [5-8]. In most healthcare systems, neonatal care is delivered by specialized professionals. While this ensures medical expertise, it may inadvertently limit parental involvement and leave caregivers feeling unprepared for discharge. Increasingly, bonding and attachment theories have influenced a shift in attitudes toward parental roles in neonatal care.

Family integrated care has emerged as a model to empower parents as active participants in the care of their hospitalized newborns. By providing structured education and integrating parents into care routines, FICare seeks to improve neonatal outcomes and foster parental confidence [9,10]. In resource-limited settings like Rwanda, parental involvement has long been a necessity rather than a formalized model of care. Due to critically low nurse-to-neonate ratios, parents are often engaged in basic caregiving tasks such as feeding, cleaning, and comforting their infants [11-14]. There is growing recognition that supporting and expanding parental roles could ease the burden on overstretched healthcare providers, improve care delivery, and promote better outcomes [13,14]. Despite this, there has been limited research in Rwanda exploring how parents experience their roles within neonatal units, their attitudes toward extended caregiving responsibilities, or the barriers they face in assuming such roles. Most existing studies have focused on healthcare provider perspectives or clinical outcomes, with little attention paid to the voices of caregivers themselves infants [11,15]. This study aimed to explore the lived experiences, attitudes, and perceptions of caregivers regarding parental care roles in two Rwandan neonatal units.

## Methods

**Study design and qualitative approach:** this was a qualitative study using semi-structured, face-to-face interviews to explore the lived experiences and perceptions of caregivers in two neonatal units in Rwanda. A pragmatic approach was used, with no guiding theoretical framework. The study followed an inductive thematic analysis strategy to allow themes to emerge from the data. Reporting adhered to the COREQ checklist for qualitative studies [16].

**Setting and study sites:** the study was conducted in two public hospitals located in Kigali, the capital city of Rwanda. The University Teaching Hospital of Kigali (CHUK) and Muhima District Hospital (MDH), were purposefully selected to capture a range of parental experiences across different levels of neonatal care. University Teaching Hospital of Kigali is one of Rwanda´s four government tertiary referral and teaching hospitals. It provides specialized care, including neonatal services, and serves as the primary teaching site for the University of Rwanda´s medical school. With an estimated 2,000 deliveries per year, the hospital´s neonatal unit admits approximately 28% of live-born infants, most of whom are referred from within the hospital or other facilities for specialized care. The unit is staffed by pediatricians, residents, and registered nurses, although only a minority of the nurses have specialized neonatal training. The unit has a capacity of 20-30 cots and operates at a level equivalent to Level II care under American Academy of Pediatrics standards [17]. University Teaching Hospital of Kigali was selected as a study site because it represents a higher-resource, higher-acuity referral setting within Rwanda´s public health system. Muhima District Hospital (MDH) is a secondary-level government hospital serving both urban and peri-urban populations in Kigali. It oversees ten affiliated health centers and manages many deliveries annually-estimated between 6,000 and 10,000. The neonatal unit has a cot capacity of 30-35 and is managed primarily by general nurses, with pediatric oversight provided by four pediatricians and periodic mentorship from a CHUK neonatologist. The unit offers basic neonatal care and is categorized as providing Level I services [17]. While high-risk cases are transferred to CHUK, MDH handles the bulk of routine neonatal admissions in the district. Both hospitals serve patients enrolled in Rwanda´s national community-based health insurance scheme (“Mutuelle de Santé”), which ensures broad accessibility to neonatal services across socio-economic groups. These two facilities were chosen for their contrasting levels of care and their capacity to reflect a diverse set of caregiver experiences from both tertiary and district-level neonatal units.

**Sampling strategy:** purposive sampling was used to capture a range of caregiver experiences. The sample included parents or female caregivers of neonates born at 28-32 weeks of gestation and admitted to either neonatal unit between January and March 2018. This gestational window was selected due to the high likelihood of prolonged hospitalization. Caregivers of neonates with a poor prognosis (e.g., major congenital anomalies or severe Hypoxic-Ischemic Encephalopathy) were excluded to minimize emotional distress.

**Data collection methods:** data collection was conducted through in-depth, semi-structured interviews with caregivers, using a topic guide developed specifically for this study. The guide was informed by qualitative research principles and adapted from Green *et al*. and it was piloted with one caregiver to assess clarity and flow [18]. The interviews were conducted in Kinyarwanda, the national language of Rwanda, by the principal investigator (AM1), a female pediatric resident who had no prior relationship with participants and was not involved in their clinical care. This approach aimed to minimize social desirability bias and create a neutral space for open dialogue. All interviews took place in a quiet, private room located near the neonatal units to ensure confidentiality and comfort. Two participants chose to have a family member present during the interview, which was permitted to support participant well-being. Interviews lasted between 30 and 60 minutes, with an average duration of 45 minutes. They were audio-recorded using two password-protected smartphones to prevent data loss due to technical failure. During each interview, field notes were taken to capture non-verbal cues and contextual information. The interviewer encouraged participants to speak freely about their experiences and perceptions related to their care roles, using open-ended questions that allowed for probing when new or significant topics emerged. Interviews were not repeated, and transcripts were not returned to participants for validation. However, iterative engagement with the data helped to refine the themes and ensure accurate representation of participant views.

**Researcher characteristics and reflexivity:** the interviewer (AM1) was a female pediatric resident with four years of neonatal experience but no prior qualitative research training. She was supported by two supervisors: PC, a pediatrician trained in the UK with qualitative research experience in Rwanda and the UK, and AM2, a Rwandan pediatrician and qualitative researcher with a PhD from Uppsala University [19,20]. The team´s clinical experience may have influenced analysis, but ongoing reflexivity and team discussions were used to mitigate bias.

**Trustworthiness and rigor:** trustworthiness was enhanced through iterative coding, peer debriefing between AM1 and PC, and transparency in documenting decisions about theme development. Though transcripts were not returned to participants, the bilingual translation and researcher´s dual reading minimized misinterpretation.

**Data processing and analysis:** audio recordings were transcribed and translated into English by a bilingual research assistant. The PI reviewed all transcripts. Thematic content analysis followed four steps: familiarization with the data, identifying and coding meaning units, organizing codes into themes, and final synthesis [21]. Data were coded inductively using Microsoft Excel. Coding was iterative and de novo, performed line-by-line by the PI. After thesis submission, coding was reviewed by PC to enhance rigor. Saturation was defined as the point at which no new codes emerged during analysis [22].

**Ethical considerations:** this study received ethical approval from the University of Rwanda Institutional Review Board (Ref: 269/CMHS IRB/2018) and the CHUK Ethics Committee (Ref: EC/CHUK/695/2018). Informed written consent was obtained from all participants after a full verbal explanation in Kinyarwanda. Participation was voluntary, and no incentives were offered. Confidentiality was maintained by anonymizing transcripts and securing data on password-protected devices. Although discussing neonatal hospitalization posed potential emotional risks, interviews were conducted with sensitivity in private spaces. No participants reported emotional distress or required referral for additional support.

## Results

A total of sixteen caregivers participated in the study, including fourteen mothers, one father, and one maternal aunt. The mean caregiver age was 24.4 years (SD ±6.3), ranging from 20 to 40 years. Most were married (87.5%) and had limited formal education, with 75% having only primary education or none. Socioeconomic status varied, with most participants falling into Rwanda´s Ubudehe category 2 (middle income), and others distributed across categories 1 (poor) and 3 (rich). Ten participants were recruited from CHUK (62.5%) and six from Muhima District Hospital (37.5%). Their neonates had a mean gestational age of 29.5 weeks (SD ±1.45), birth weight of 1205 grams (SD ±285), and were, on average, 19.8 days old (SD ±16.4) at the time of interview. Full sociodemographic characteristics are presented in Table 1. Thematic analysis revealed seven overarching themes, summarized in Table 2 and described below. Specific care tasks are detailed in Table 3.

**Table 1:** background characteristics of caregivers and neonates

Characteristic	Category	n=16
**Relationship to neonate**	Mother	14 (88%)
	Father	1 (6%)
	Aunt	1 (6%)
**Caregiver age (years)**	Mean (SD†)	24.4 (±6.3)
**Marital status**	Married	14 (87.5%)
	Single	2 (12.5%)
**Level of education**	None or Primary	12 (75%)
	Secondary or University	4 (25%)
Socioeconomic status (Ubudehe**)	Category 1 (poor)	2 (13.3%)
	Category 2 (middle)	9 (60%)
	Category 3 (rich)	3 (20%)
	Unknown	1 (6.6%)
**Hospital site**	CHUK†	10 (62.5%)
	Muhima District Hospital	6 (37.5%)
Neonate gestational age (weeks)	Mean (SD)	29.5 (±1.45)
Birth weight (grams)	Mean (SD)	1205 (±285)
Age of neonate at interview (days)	Mean (SD)	19.8 (±16.4)

* SD: Standard deviation; **Ubudehe categories is a socioeconomic system that previously classified Rwandan households based on their socioeconomic status: category 1 (extremely poor/indigent), category 3 (poor), category 3 (middle), category 4 (richer), and category 5 (richest). In this study, the first two categories were condensed into category 1 (poor), and the last two categories into category 3 (rich), with the middle category becoming category 2 (middle). †CHUK: University Teaching Hospital of Kigali

**Table 2:** summary of themes and representative quotes

Theme	Sub-theme	Illustrative quote
**Emotional experiences**	Anxiety and helplessness	*I do not know when I will be discharged… I am anxious* (Interview 2)
	Fear and sadness	*I get stressed when the baby is sick* (Interview 5)
	Shock and depression	*I feel so depressed when I see him naked* (Interview 15)
**Existing caregiving roles**	Feeding and hygiene	*I clean, wash and feed him with so much love* (Interview 12)
	Bonding through interaction	*I talk to him and sing for him* (Interview 8)
	Providing basic care	*I can give breast milk in the NGT*†† (Interview 16)
**Readiness to provide care**	Prepared with support	*As we were educated by the medical staff, I became confident* (Interview 12)
	Lack of readiness	*It is not easy… I have not yet recovered from difficult labour* (Interview 10)
**Attitudes toward extended roles**	Willingness to do more	*They could teach me how to give oral medication* (Interview 14)
	Reluctance without HCP' support	*I cannot do anything without doctor's help* (Interview 6)
**Perceived benefits of involvement**	Stronger bond	*He could feel my love so much* (Interview 8)
	Confidence and readiness for discharge	*I could know how to take care of my child without any guard* (Interview 3)
**Barriers to care**	Maternal illness	*I cannot do anything for them because I am sick* (Interview 13)
	Limited access to unit	*When someone does not open the door, this may lead to late feeding* (Interview 11)
	Resource constraints	*I do not have enough breastmilk* (Interview 2)
**Support needed**	Training and education	*The help from them would be approaching me and discussing, so I learn how to care* (Interview 3)
	Communication and updates	*I would like to have explanations about the progress of my baby* (Interview 1)
	Financial assistance	*They should avail medications according to the money we have* (Interview 9)

† †NGT: Nasogastric tube. HCP: Healthcare provider(s)

**Table 3:** specific care tasks reported by caregivers

Care task	Frequency reported (n=16)	Illustrative comment
Diaper changing	15	*I change his diapers myself*
Bathing and cleaning	14	I bathe him with so much love.
Tube feeding	12	*I can give breast milk in the NGT*††
Skin-to-skin care/bonding	11	*I talk to him and sing for him*
Breastfeeding	12	I breastfeed when they bring the baby to me
Giving oral medication	5	*They could teach me how to give oral medication*
Checking stool/hygiene	9	*I check if the baby is dirty and clean him if needed*
Washing baby clothes	10	*I wash his clothes and keep everything clean*
Applying lotion	7	*I put lotion on his skin after bathing him*
Monitoring temperature	4	*I would like to learn how to check his temperature myself*

† †NGT: Nasogastric tube

**Theme 1: emotional experiences in the neonatal unit:** caregivers described the neonatal environment as emotionally overwhelming, particularly during the early days of hospitalization. Many expressed anxiety, helplessness, and sadness when observing their infants in incubators or when dealing with medical equipment. For example, one parent noted: *“Personally, I feel so depressed when I always see him naked. I want him to wear clothes*.” (interview 15). Others expressed worry about not knowing their baby´s condition or discharge timeline: *“I do not know when I will be discharged… I am anxious*.” (interview 2). Some caregivers drew strength from religious faith or peer support, which helped them cope with emotional stress over time. Familiarity with the unit, guidance from healthcare professionals, and bonding with their baby gradually improved their emotional state.

**Theme 2: existing caregiving roles:** parents were already engaged in a variety of caregiving tasks such as cleaning, diaper changing, tube feeding, skin-to-skin bonding, and comforting their babies through touch and speech. These roles, though informal, were often essential in under-resourced units with limited staff. One mother stated: *“I clean, wash and feed him with so much love*.” (interview 12), while another shared: *“I talk to him and sing for him.”* (interview 8). These caregiving practices reflected both necessity and cultural norms that emphasize maternal responsibility and emotional closeness.

**Theme 3: readiness to provide care:** most caregivers expressed a progressive increase in confidence as they received informal instruction from staff. *“As we were educated by the medical staff, I became confident, and I started taking care of my baby*.” (interview 12). Readiness was often linked to time spent in the unit, observational learning, and trust developed with healthcare providers. However, some noted that physical recovery after childbirth posed limitations to their readiness, stating, *“It is not easy… I have not yet recovered from difficult labour*.” (interview 10). Others mentioned that anxiety and lack of prior experience created initial hesitation, although this often reduced with time and support.

**Theme 4: attitudes toward extended caregiving roles:** several caregivers welcomed the opportunity to expand their responsibilities to include weighing their infants, checking temperature, and administering oral medication, as long as training was provided. As one stated: *“They could teach me how to give oral medication and how to take weight.”* (interview 14). Parents expressed interest in learning tasks that were not overly medicalized or invasive, preferring those perceived as extensions of maternal care. A few were hesitant, preferring to rely on healthcare professionals for more complex or unfamiliar care, especially when their infants were critically ill. *“I cannot do anything without doctor´s help.”* (interview 6).

**Theme 5: perceived benefits of caregiver involvement:** parents highlighted that active participation enhanced bonding, fostered confidence, and promoted emotional well-being. They also felt that it supported infant development and reduced hospital-acquired infections by ensuring better hygiene and prompt care. One caregiver said: *“If I am the one who provides care… I could know him better.”* (interview 2). Many believed that their involvement would make the transition to home care smoother and improve their competence post-discharge. Others acknowledged that their participation helped ease the workload of overwhelmed staff and contributed to more personalized attention for their babies.

**Theme 6: barriers to providing or extending care:** barriers included maternal illness, fatigue, emotional stress, and logistical constraints like locked units and lack of supplies. Additionally, caregivers cited inadequate communication with healthcare providers and financial challenges. *“When someone does not open the door, this may lead to late feeding of the child.”* (interview 11). Some mothers explained they had limited breast milk or could not afford nutritious food and medications, impacting their ability to care. These constraints were often compounded by restricted access to infants or unclear guidelines on parental roles within the unit.

**Theme 7: support needs to enable extended care:** participants requested clearer communication, consistent clinical updates, emotional support, and structured training. *“The help from them would be approaching me and discussing, so I learn how to care for the child.”* (interview 3). They also emphasized the need for empathy, encouragement, and a welcoming environment where their questions and efforts were acknowledged. Material needs such as food, baby clothes, and hygienic supplies were also mentioned, along with requests for affordable medication and better sleeping arrangements for mothers staying near the unit. Themes, sub-themes, and representative quotes are presented in Table 2. Detailed accounts of caregiving tasks appear in Table 3.

## Discussion

This study aimed to explore the lived experiences, attitudes, and perceptions of caregivers regarding their roles in neonatal care in two public hospitals in Rwanda. Through thematic analysis of sixteen in-depth interviews, seven key themes were identified: emotional experiences in the neonatal unit, existing caregiving roles, readiness to provide care, attitudes toward extended caregiving roles, perceived benefits, barriers to participation, and support needs. These findings provide new insight into caregiver perspectives in the context of resource-limited neonatal units. Parents consistently reported emotional distress associated with their newborn´s hospitalization, especially during the initial period of adapting to an unfamiliar environment, complex equipment, and the clinical instability of their babies [5-8]. These findings are in line with other studies in sub-Saharan Africa and globally, which have documented parental anxiety, sadness, and uncertainty as typical reactions to neonatal intensive care settings [11,15,23]. Yet, despite these emotional challenges, parents in our study were already actively engaged in their babies´ care-undertaking tasks such as hygiene, feeding, and bonding. Their engagement often resulted not from formal training, but from necessity due to high patient-to-nurse ratios. This informal involvement aligns with Family Integrated Care models [9,10], which aim to integrate parents into neonatal care more formally. However, in our setting, parental participation developed organically in response to systemic limitations. Our findings suggest that, with appropriate support and education, these existing informal roles could be developed into structured, safe, and empowering care practices [11,12,24].

Participants expressed a high level of willingness to take on extended care roles such as monitoring temperature, administering oral medications, and weighing their infants. These intentions were often motivated by a desire to bond, understand their baby´s condition, and feel more prepared for discharge. In contrast to prior findings from a rural Rwandan neonatal unit where parents appeared less willing to assume new tasks [11], our urban-based sample may reflect differing levels of education, exposure, and access to health information. The discrepancy also underscores the need to contextualize parental engagement within sociocultural and infrastructural realities. Importantly, caregivers recognized that their deeper involvement could benefit not only their babies but also the healthcare system by reducing the burden on overextended healthcare professionals. However, several barriers to effective participation were identified, including caregivers´ physical recovery post-delivery, limited access to the neonatal unit, lack of knowledge or skills, and inadequate communication with healthcare professionals [25-33]. These challenges reflect structural issues within the healthcare system, such as insufficient staffing, unclear protocols for parent-provider communication, and financial constraints, all of which can diminish caregiver confidence and engagement. Parents repeatedly emphasized the need for structured education, emotional support, and consistent communication to empower them to assume more responsibilities safely. These findings mirror international evidence that comprehensive orientation and training improve parental confidence and neonatal outcomes [23,27,34,35].

Additionally, low-cost interventions like peer support from “veteran mothers,” targeted visual education tools (e.g., Global Health Media videos) [36], and improved caregiver access to facilities (e.g., avoiding locked unit doors) could enhance caregiver readiness and wellbeing. While many caregivers were enthusiastic about greater involvement, some voiced concern that their role might overlap or conflict with professional responsibilities. As other studies have shown [35,37], expanding family-centered models may risk tension between staff and caregivers unless role expectations are clearly communicated and supported institutionally. Our study also highlights that parental participation should not be viewed as a replacement for trained professionals but rather as a complementary and supportive model. The findings of this study have several implications for neonatal care in low-resource settings. First, they underscore the importance of recognizing and formalizing the informal caregiving roles that already exist in many neonatal units. Rather than viewing parental care as an auxiliary function, healthcare systems should integrate parents as active partners in neonatal care through policy, training, and infrastructure. This approach could not only improve the quality of care but also promote better developmental and psychological outcomes for neonates and their families. Second, the study highlights the need for targeted caregiver education, delivered through low-cost, scalable strategies such as peer mentorship, group orientation, and visual tools. Finally, institutional policies should be adapted to facilitate caregiver access to neonatal units, ensure clear role boundaries between professionals and parents, and promote collaborative, respectful relationships between caregivers and healthcare providers. This study has several strengths. It offers in-depth, first-hand insights from caregivers in two different levels of care settings and applies a rigorous qualitative approach to data collection and analysis. However, it also has limitations. The use of individual interviews, while allowing for detailed exploration, may have constrained the diversity of viewpoints that might have emerged in focus groups. Translation from Kinyarwanda to English introduces potential for loss of nuance, though efforts were made to minimize this through close review by the bilingual interviewer. Social desirability bias may have influenced some responses, given the interviewer's clinical background.

## Conclusion

Caregivers in two urban neonatal units in Rwanda described a strong willingness to take on extended roles in caring for their hospitalized newborns, motivated by emotional, cultural, and practical factors. Their participation was shaped by existing health system constraints and a desire to be more involved. To support and safely expand these roles, structured caregiver education, emotional support, improved access, and clear communication with healthcare providers are essential. Integrating parents into neonatal care in low-resource settings requires thoughtful implementation, but has the potential to benefit both families and overburdened healthcare systems.

### What is known about this topic

- Parents often experience stress and anxiety when their newborns are admitted to neonatal intensive care units;
- Family integrated care improves outcomes by involving parents in neonatal care;
- In low-resource settings, parental involvement is common but often informal and poorly supported

### What this study adds

- Rwandan caregivers are already engaged in essential neonatal care activities due to systemic staffing constraints;
- Caregivers are willing to take on extended care roles if supported with training, access, and communication;
- Structural and emotional support is necessary to formalize and safely expand parental roles in neonatal units.
